# PGPR Reduce Root Respiration and Oxidative Stress Enhancing *Spartina maritima* Root Growth and Heavy Metal Rhizoaccumulation

**DOI:** 10.3389/fpls.2018.01500

**Published:** 2018-10-17

**Authors:** Jennifer Mesa-Marín, Néstor Fernández Del-Saz, Ignacio D. Rodríguez-Llorente, Susana Redondo-Gómez, Eloísa Pajuelo, Miquel Ribas-Carbó, Enrique Mateos-Naranjo

**Affiliations:** ^1^Departamento de Biología Vegetal y Ecología, Facultad de Biología, Universidad de Sevilla, Seville, Spain; ^2^Grup de Recerca en Biologia de les Plantes en Condicions Mediterrànies, Universitat de les Illes Balears, Palma, Spain; ^3^Departamento de Botánica, Facultad de Ciencias Naturales y Oceanográficas, Universidad de Concepción, Concepción, Chile; ^4^Departamento de Microbiología y Parasitología, Facultad de Farmacia, Universidad de Sevilla, Seville, Spain

**Keywords:** plant growth promoting rhizobacteria (PGPR), heavy metals, *Spartina maritima*, root respiration, oxygen-isotope fractionation, oxidative stress, carbon balance, bioremediation

## Abstract

The present study aims to unravel ecophysiological mechanisms underlying plant-microbe interactions under natural abiotic stress conditions, specifically heavy metal pollution. Effect of plant growth promoting rhizobacteria (PGPR) bioaugmentation on *Spartina maritima in vivo* root respiration and oxidative stress was investigated. This autochthonous plant is a heavy metal hyperaccumulator cordgrass growing in one of the most polluted estuaries in the world. The association with native PGPR is being studied with a view to their biotechnological potential in environmental decontamination. As a novelty, the oxygen-isotope fractionation technique was used to study the *in vivo* activities of cytochrome oxidase (COX) and alternative oxidase (AOX) pathways. Inoculated plants showed decreased antioxidant enzymatic activities and *in vivo* root respiration rates. The reduction in respiratory carbon consumption and the stress alleviation may explain the increments observed in *S. maritima* root biomass and metal rhizoaccumulation after inoculation. For the first time, plant carbon balance and PGPR are interrelated to explain the effect of rhizobacteria under abiotic stress.

## Introduction

Heavy metal pollution in soil is one of the most serious ecological problems all over the world, as it causes negative impacts on the ecosystem and plant, animal and human health (Vassilev et al., 2004; Nagajyoti et al., 2010; Ali et al., 2013). Heavy metals cannot be degraded to harmless products, and consequently persist in the environment indefinitely (Garbisu and Alkorta, 2001). Many remediation strategies have been considered to counter the detrimental effects of metal excess in soil (Sharma and Pandey, 2014). Among them, using metal accumulating plants has been receiving increasing attention due to their good potential for success (Burd et al., 2000).

Spartina maritima (Curtis) Fernald is an indigenous cordgrass that naturally grows in the joint estuary of Tinto and Odiel rivers (SW Spain), one of the most polluted areas by heavy metals in the world (Mesa et al., 2016). This C4 halophyte is an important salt marsh pioneer and ecosystem engineer (Mateos-Naranjo et al., 2010) that possesses a high heavy metal accumulating capacity in tissues, especially in its roots, making it useful for phytoremediation purposes (Redondo-Gómez, 2013). Despite the fact that metal hyperaccumulator plants like *S. maritima* can resist pollution to certain degree, it is well known that exposure to heavy metals triggers a wide range of physiological and biochemical alterations in plants (Singh et al., 2015), ultimately leading to reduced growth and metal phytoaccumulation (Duarte et al., 2013). In this context, the association with plant growth promoting rhizobacteria (PGPR) plays an important role toward enhancement of plant development under heavy metal stress, thus ameliorating phytoremediation capacity of hyperaccumulator plants (Glick, 2010; Rajkumar et al., 2012).

Improvement of *S. maritima* root growth and metal rhizoaccumulation after PGPR inoculation in polluted sediments has been recently demonstrated (Mesa et al., 2015a). This positive effect was mainly mediated by improvement of photosynthetic apparatus performance, linked with a beneficial impact on PSII functionality and chlorophyll concentration. However, despite amelioration in leaf fitness, the bacterial consortium was able to stimulate plant growth and metal uptake chiefly in roots (Mesa et al., 2015a). Taking into consideration that plant growth depends on the accumulation of photosynthetic carbon not consumed during respiration (Ribas-Carbó et al., 2000; González-Meler et al., 2001; Lambers et al., 2008; Del-Saz et al., 2016; Flórez-Sarasa et al., 2016), it is reasonable to study both photosynthesis and respiration processes in *S. maritima* in order to elucidate the manner by which plant growth is increased under PGPR inoculation. With this in mind, it is known that high concentrations of heavy metals greatly alter respiration in plants (Lösch, 2004) by affecting different components of the mitochondrial electron transport chain (mETC) (Keunen et al., 2011). Among them, alternative oxidase (AOX) and cytochrome oxidase (COX) have been, by far, the most studied enzymes of the mETC (Vanlerberghe, 2013). In particular, AOX is thought to play an important role under the stress induced by metal toxicity (Keunen et al., 2011). However, there is no information about the response of the *in vivo* activities of AOX and COX pathways under metal excess and the possible effects of PGPR inoculation on plant respiration under heavy metal stress. Thus, we hypothesize that previously observed beneficial root effect after bacterial bioaugmentation may be linked to a positive carbon balance at root level. It is plausible that microbial inoculation may alter COX and AOX pathways. Indeed, the scales may be tilted in favor of a greater relative effect on AOX component, due to its significance in plant response against stress that comes with heavy metal exposure. Moreover, it should not be forgotten that other defensive responses, including the activities of antioxidant enzymes that reduce reactive oxygen species (ROS) levels, such as catalase (CAT), guaiacol peroxidase (GPX), and superoxide dismutase (SOD) activate under metal stress, and are also susceptible of bacterial regulation (Dimkpa et al., 2009a,b; Das and Roychoudhury, 2014).

The present study links two fields that are rarely combined: the bioremediation/PGPR literature and the respiratory physiology/carbon balance literature. It aims at describing the ecophysiological response, at the organism level, of the autochthonous cordgrass *S. maritima* after PGPR bioaugmentation in natural heavy metal polluted sediments, with a special emphasis on *in vivo* root respiration and oxidative stress. It should provide a new insight into our understanding of plant biology in the context of PGPR-associated phytoremediation, with a view to the biotechnological potential of hyperaccumulator plants in environmental decontamination.

## Materials and Methods

### Plant and Soil Source and Growing Conditions

In June 2016, 10 cm diameter clumps of *S. maritima* were randomly collected from a natural population located in a well-drained gently sloping intertidal low-marsh (mean sea level + 1.30 m relative to Spanish Hydrographic Zero, SHZ) from the Tinto river salt marsh (37°15′N, 6°58′W; SW Spain). Clumps were planted in individual plastic pots (15 cm high × 18 cm diameter), filled with 1 kg of soil from the marsh and placed in a glasshouse with temperatures of 21–25°C, 40–60% relative humidity, natural day light and irrigated with tap water. Pots were kept under these conditions for 1 week and then were randomly assigned to two bioaugmentation treatments (details in the next section). Tap water metal concentrations were: arsenic (As) < 1 μg l^-1^, cadmium (Cd) < 1 μg l^-1^, copper (Cu) < 0.01 mg l^-1^, nickel (Ni) < 5 μg l^-1^, lead (Pb) < 5 μg l^-1^ and zinc (Zn) < 0.01 mg l^-1^. Tinto sediment psychochemical properties are given in **Table 1**, which also shows metal threshold values imposed for remedial action.

**Table 1:** Concentration of arsenic (As), cadmium (Cd), copper (Cu), nickel (Ni), lead (Pb), and zinc (Zn) and physicochemical properties of sediments from Tinto marshes.

	Metal concentration (mg Kg^-^^1^)											
		As		Cd	Cu		Ni	Pb		Zn
Tinto sediments		524 ± 31		4.6 ± 0.4	2968 ± 211		34.3 ± 1.5	610 ± 38		2576 ± 192
Nature park soil^2^		>100		>15	>500		>500	>1000		>1000
Agricultural soil^2^		>50		>7	>300		>200	>350		>600

**Physico-chemical properties**											

**Texture^1^**	**pH**		**Redox potential (mV)**			**Conductivity (mS cm**^-^**^1^)**			**Organic matter (%)**	

71/19/10	6.3 ± 0.2		195 ± 12			12.6 ± 0.5			11.5 ± 0.6	

*Values are means ± SE (*n* = 6). ^*1*^Texture (silt/clay/sand percentage). ^*2*^Reference threshold values stablished by the local Government to measure pollution severity and to guide corrective actions. Beyond these intervention values, remedial action must be taken in the soil (Junta de Andalucía Consejería de Medio Ambiente, 1999)*.

### Bacterial Strains, Inoculant Solution, and Bioaugmentation Treatment

Bacteria used in this work were isolated from the rhizosphere of *S. maritima* grown in the Tinto river estuary, SW Spain (Mesa et al., 2015a). They were identified by PCR amplification and sequencing of the 16S rDNA as *Bacillus methylotrophicus* SMT38 (Accession No. KF962966), *Bacillus aryabhattai* SMT48 (Accession No. KF962976), *Bacillus aryabhattai* SMT50 (Accession No. KF962978), and *Bacillus licheniformis* SMT51 (Accession No. KF962979) (Mesa et al., 2015a). The resistance of these bacteria to different heavy metals and NaCl was determined on plates containing TSA 0.2 M NaCl medium (according to sediment conductivity), both supplemented with increasing concentrations of heavy metals or NaCl from stock solutions (Mesa et al., 2015a). These bacteria showed a high resistance to several heavy metals and metalloids (up to 10 mM Cu, 4 mM Zn, 18 mM As or 20 mM Pb), as well as to NaCl (up to 2 M NaCl). Moreover, these bacteria exhibited multiple plant growth promoting properties, such as nitrogen fixation, phosphate solubilisation, biofilm-forming capacity and production of siderophores and indole-3-acetic acid, demonstrated by several screening tests for plant growth promoting traits (Mesa et al., 2015a). Finally, the four bacterial isolates were cultivated together and none of them showed antagonistic activity against each other (data not shown). To prepare the inoculant solution, bacteria were grown separately in 250 ml Erlenmeyer flasks containing 50 ml of TSB 0.2 M NaCl medium and incubated under continuous gentle shaking at 28°C during 18 h. Then, cultures were centrifuged in 50 ml Falcon tubes at 8000 rpm during 10 min and the supernatant was discarded. Pellets were washed twice with tap water and finally resuspended in tap water to get a suspension with an OD_600_ of 1.0 (ca. 10^8^ cells per ml). Then, equal amounts of the four bacterial suspensions were mixed to get the final inoculant solution. Pots were randomly assigned to two treatments (*n* = 12, 6 pots in each one): control non-inoculated plants and inoculated plants. During the assay, pots were slightly watered with tap water every 2 days. For plant inoculation, every pot was watered with 10 ml of the inoculant solution (ca. 10^9^ cells per pot) at the beginning of the experiment (Mesa et al., 2015b).

### Plant Biomass and Ions Concentration in Plant Tissues

At the end of the experiment, 30 days after treatment initiation, plants were harvested and separated into roots and shoots and dried at 60°C for 48 h before weighing (*n* = 6).

For tissues ions concentration, leaf and root samples were randomly collected and successively washed with distilled water in order to remove ions from the free spaces and from its surface prior to analysis (*n* = 6). After that, leaf and root samples were ground as previously described in Mateos-Naranjo et al. (2008) and digested with 6 ml HNO_3_, 0.5 ml HF and 1 ml H_2_O_2_ at 130°C for 5 h in triplicate. Then, As, Cd, Cu, Ni, Pb, and Zn were measured by inductively coupled plasma (ICP-OES) spectroscopy (ARL-Fison 3410, United States). For quality control, the accuracy and precision of analytical procedure was checked every 5 samples by routine determination of total element concentrations using reference materials from Fisons certified. The average of uncertainty in the determination of elements was in all cases < 2%.

### *In vivo* Root Respiration and Oxygen-Isotope Fractionation Measurements

Respiration and oxygen isotope fractionation measurements were performed in randomly selected root samples (approximately 300 mg fresh weight, FW) of five replicate plants 1 day before complete plants were harvested for growth analysis.

Roots samples were immediately carefully rinsed using a soft water jet and left to air dry during 15 min before to be placed in a 3 ml stainless-steel closed cuvette maintained at a constant temperature of 25°C (Gastón et al., 2003). The respiration cuvette was equipped with two inlets: one connected to the mass spectrometer (Delta XPlus, Thermo LCC, Bremen, Germany), and the other connected to a 2 ml air-tight syringe. Throughout the experiment the syringe was used to both mix the air in the cuvette and to maintain the cuvette at constant pressure. Air samples of 300 μl were sequentially withdrawn from the cuvette and fed into the mass spectrometer. Changes in the ^18^O/^16^O ratios and oxygen concentration were obtained to calculate the oxygen-isotope fractionation and respiration rates (Ribas-Carbó et al., 2005). The electron partitioning to the alternative pathway (τ_a_) was calculated as follows:

(1) $$\tau_{a}=(\Delta_{n}-\Delta_{\text{c}})/(\Delta_{\text{a}}-\Delta_{\text{c}})$$

Where Δc, Δa are the oxygen-isotope fractionation of the cytochrome (+ SHAM) and alternative (+ KCN) pathway, respectively, and Δ_n_, is the oxygen-isotope fractionation of the respiration in the absence of inhibitors. For Δ_a_ measurements, roots were submerged in a solution of 10 mM KCN for 30 min. In addition, a piece of medical wipe wetted with 10 mM KCN was placed in the cuvette. A value of Δ_a_ of 28.03 ± 0.03‰ (*n* = 3) was obtained. For the calculation of Δ_c_, roots were submerged in freshly solutions of 25 mM SHAM for 30 min. A value of Δ_c_ for 18.0 ± 0.06‰ (*n* = 3) was obtained.

The individual activities of the cytochrome oxidase pathway, COP (*v*_cyt_) and AOX pathway, AOP (*v*_alt_) were obtained by multiplying the total oxygen uptake rate (V_t_) and the partitioning to each pathway as follows:

$$\begin{array}{l} \nu_{cyt}=V_{\text{t}}\times (1-{\text{τ}}_{\text{a}})\\ \nu_{\text{alt}}=V_{\text{t}}\times {\text{τ}}_{\text{a}} \end{array}$$

### Antioxidant Enzymes Assays

Enzyme extraction was done following the methodology used by Duarte et al. (2015). At the end of experiment, 500 mg of fresh roots and leaf samples were grounded in 8 ml of 50 mM sodium phosphate buffer (pH 7.6) with 0.1 mM Na-EDTA and were centrifuged at 10,000 × *g* for 20 min at 4°C to obtain the soluble proteins. Five samples per inoculation treatment were used and three measurements per sample were registered. Catalase (CAT; EC1.11.1.6) activity was measured according to Teranishi et al. (1974), by monitoring the consumption of H_2_O_2_ and consequent decrease in absorbance at 240 nm (𝜀 = 39.4 mM^-1^ cm^-1^). The reaction mixture contained 50 mM of sodium phosphate buffer (pH 7.6), 0.1 mM of Na-EDTA and 100 mM of H_2_O_2_. The reaction was started with the addition of 100 μl of enzyme extract. Guaiacol peroxidase (GPX; EC1.11.1.7) was measured by the method of Bergmeyer et al. (1974), with a reaction mixture consisting of 50 mM of sodium phosphate buffer (pH 7.0), 2 mM of H_2_O_2_ and 20 mM of guaiacol. The reaction was initiated with the addition of 100 μl of enzyme extract. The enzymatic activity was measured by monitoring the increase in absorbance at 470 nm (𝜀 = 26.6 mM^-1^ cm^-1^). Superoxide dismutase (SOD; EC1.15.1.1) activity was assayed according to Marklund and Marklund (1974) by monitoring the reduction of pyrogallol and the increase of absorbance at 325 nm. The reaction mixture contained 50 mM of sodium phosphate buffer (pH 7.6), 0.1 mM of Na-EDTA, 3 mM of pyrogallol and Mili-Q water. The reaction was started with the addition of 10 μl of enzyme extract. One enzyme activity was defined as the amount of enzyme capable of inhibiting 50% of the autoxidation of pyrogallol. Control assays were done in the absence of substrate in order to evaluate the auto-oxidation of the substrates. To calculate the enzyme activity per μg of protein, total protein content in leaf and root extracts was determined according to Bradford (1976).

### Statistical Analysis

Statistical analyses were carried out using ‘Statistica’ v. 6.0 (Statsoft Inc.). The differences between means the two inoculation treatments and between leaf and root ions and antioxidative activity at the end of the experiment were made by using one-way analysis of variance (*F*-test). Finally, Pearson coefficients were calculated between log10 transformed-fold changes of the respiratory and antioxidative variables, in order to assess correlations between them. Data were first tested for normality with the Kolmogorov–Smirnov test and for homogeneity of variance with the Brown–Forsythe test.

## Results

### *S. maritima* Growth Analysis and Ion Tissues Concentrations

**FIGURE 1:**
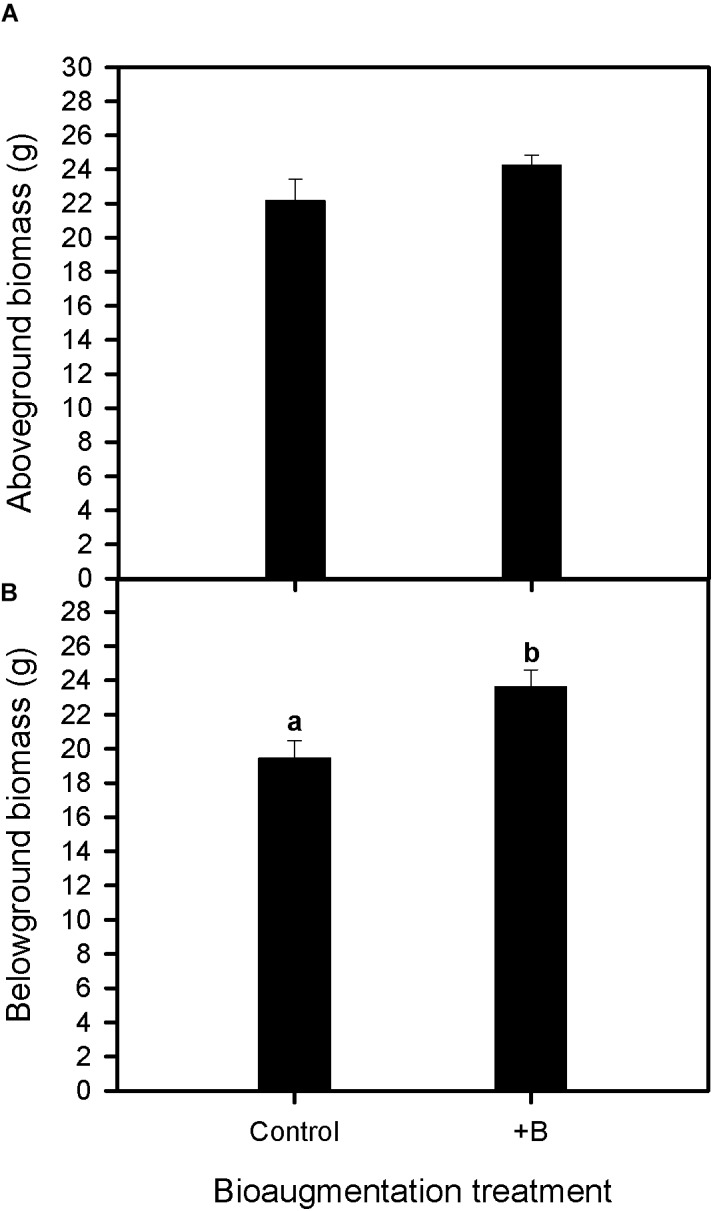
Effect of bioaugmentation with the rhizobacterial consortium (+ B) on aboveground biomass **(A)** and belowground biomass **(B)** in *Spartina maritima* plants grown in natural sediment from Tinto marsh for 30 days. Values are means ± SE (*n* = 6). Different letters indicate means that are significantly different from each other (*F*-test, *P* < 0.05).

At the end of the experiment (30 days), soil bioaugmentation with the native bacterial consortium increased the belowground biomass of *S. maritima* by about 20% (one-way Anova, *P* < 0.05), whereas no significant differences were recorded for aboveground biomass respect to plants grown without bacterial bioaugmentation (**Figure 1**).

**FIGURE 2:**
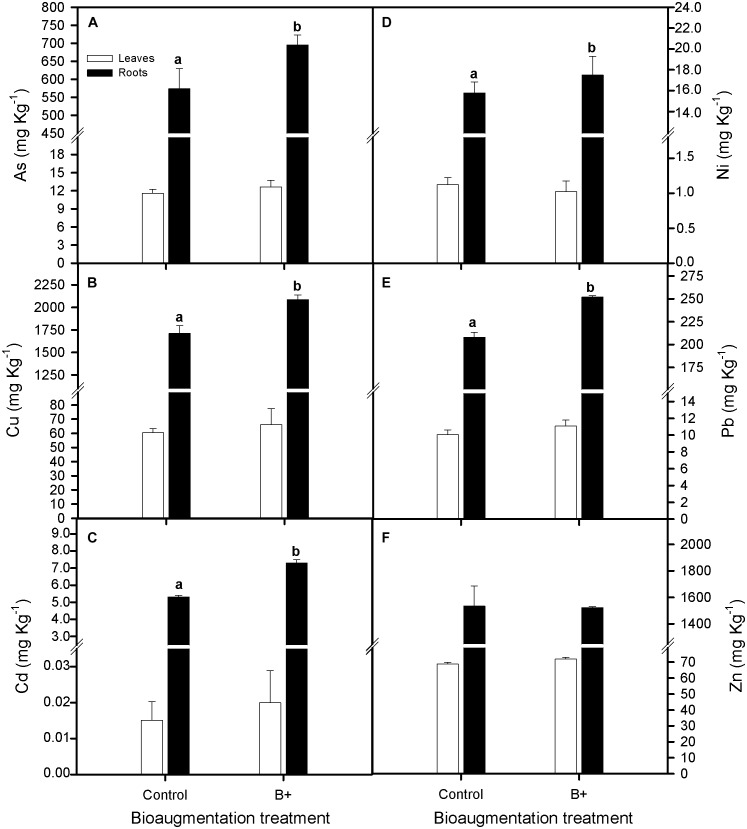
Effect of bioaugmentation with the rhizobacterial consortium (+ B) on total **(A)** arsenic, As, **(B)** copper, Cu, **(C)** cadmium, Cd, **(D)** nickel, Ni, **(E)** lead, Pb, and **(F)** zinc, Zn accumulation for leaves and roots of *Spartina maritima* grown in natural heavy metal polluted sediment from Tinto marsh for 30 days. Values are means ± SE (*n* = 6). Different letters indicate means that are significantly different from each other (*F*-test, *P* < 0.05).

On the other hand, tissue ion concentrations were greater in the roots than in leaves (One-way Anova, *P* < 0.05) and rhizoinoculation treatment favored the capacity of *S. maritima* to accumulate As, Cu, Cd, and Pb in its roots, being the increment in those ions concentrations of 21, 22, 37, and 21%, respectively (one-way Anova, *P* < 0.05; **Figures 2A–C,E**), while root and leaves Ni and Zn ions concentrations did not vary between both inoculation treatments (**Figures 2A–F**).

### *S. maritima in vivo* Root Respiration and Oxygen-Isotope Fractionation Measurements

**FIGURE 3:**
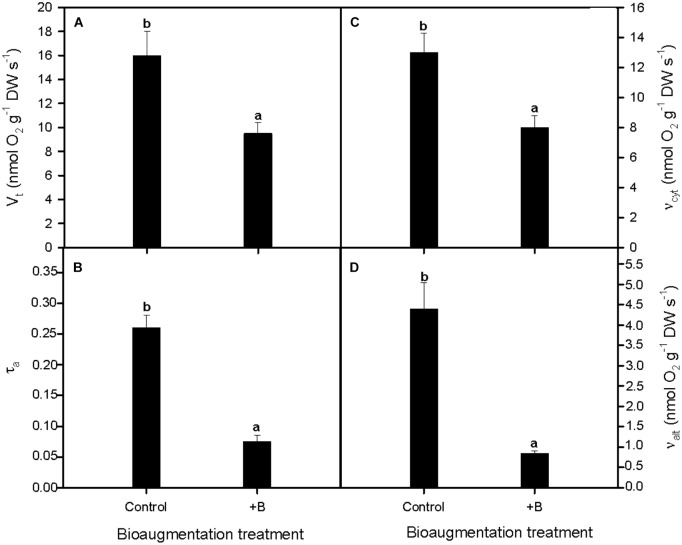
Effect of bioaugmentation with the rhizobacterial consortium (+ B) on *in vivo* root respiratory activities as determined with the oxygen-isotope fractionation technique. **(A)** Total respiration rate (*V*_t_), **(B)** electron partitioning to the alternative pathway (τ_a_), and the *in vivo* activities of **(C)** cytochrome oxidase (*v*_cyt_) and **(D)** alternative oxidase (*v*_alt_) of *S. maritima* grown in natural heavy metal polluted sediment from Tinto marsh for 30 days. Values are means ± SE (*n* = 5). Different letters indicate means that are significantly different from each other (*F*-test, *P* < 0.05).

Our results showed that total respiration rate (*V*_t_) in *S. maritima* roots decreased considerably when the rhizobacterial consortium was used (**Figure 3A**). This reduction reached a 44.1% (one-way Anova, *P* < 0.05, **Figure 3A**). The electron partitioning to the AOX pathway provided a downscaling approach of these differences between both inoculation treatments in respiratory activity. Thus, the lower *V*_t_ was accompanied by a notable diminishing of the electron partitioning to the alternative pathway (τ_a_) in inoculated plants, being this reduction of 59.2% compared with their non-inoculated counterparts (one-way Anova, *P* < 0.01, **Figure 3B**). Furthermore, it should be highlighted that respiratory activity variation was in greater extent due to higher changes in *v*_alt_ compared to *v*_cyt_. Thus, compared with non-inoculated plants, *v*_alt_ and *v*_cyt_ decreased 69.7 and 30.8% respectively (one-way Anova, *P* < 0.05; **Figures 3C,D**).

### Antioxidant Enzymes Activity

**FIGURE 4:**
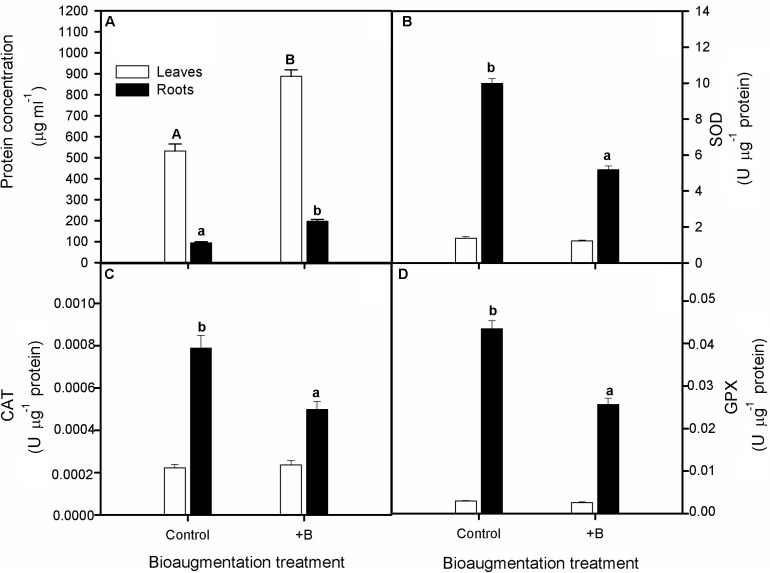
Effect of bioaugmentation with the rhizobacterial consortium (+ B) on soluble protein content **(A)**, catalase, CAT **(B)**, superoxide dismutase **(C)** and guaiacol peroxidase, GPX **(D)** enzymatic activities in leaf and roots randomly selected of *S. maritima* grown in natural heavy metal polluted sediment from Tinto marsh for 30 days. Values are means ± SE (*n* = 5). Different letters indicate means that are significantly different from each other (*F*-test, *P* < 0.05). Capital letters refer to differences in root while lowercase in leaf.

By the end of the experiment, bacterial bioaugmentation treatment increased considerably soluble protein content both in leaves and roots in S. *maritima* (one-way Anova_leaves and roots,_ *P* < 0.05; **Figure 4A**). Also, concerning the antioxidative enzymatic activity, we found that CAT, SOD, and GPX activities were higher for roots than for shoots in both inoculation treatments (One-way Anova, *P* < 0.05) and decreased significantly in roots with bioaugmentation treatment (**Figures 4B–D**). Thus, compared with non-inoculated plants, these reductions were of 37, 48, and 43% for CAT, SOD and GPX activities, respectively (one-way Anova, *P* < 0.05; **Figure 4B–D**). Contrarely, bioaugmentation treatment did not have any significative effect on enzyme activities in leaves (**Figure 4B–D**).

## Discussion

This work analyses for the first time *in vivo* root activities of COX and AOX pathways during plant respiration in a bioremediation context. As another novelty, the effects of PGPR on the antioxidative enzyme response of *S. maritima* under heavy metal stress are reported. Uncommonly, these studies were carried out under natural heavy metal stress conditions, using natural Tinto estuary sediments, and a non-model plant, given the potential of native *S. maritima* for metal phytoremediation and its utmost important role in estuarine dynamics (Mateos-Naranjo et al., 2010; Redondo-Gómez, 2013).

Bioaugmentation was the inoculation strategy followed. This is, the addition of competent microorganisms, including the reinoculation of soil with indigenous microorganisms directly isolated from the collection site. The rationale behind this approach, supported by several studies (Vogel, 1996; Thompson et al., 2005), is that a strain derived from a population that is temporally and spatially prevalent in a specific type of habitat, is more likely to persist as an inoculum when reintroduced, than one that is transient or even alien to such a habitat, much more so in polluted scenarios like Tinto saltmarsh. Presumably, these strains are already present in the rhizosphere in the uninoculated treatment, and they have demonstrated to be advantageous for plant growth (Mesa et al., 2015a). But their effect is sub-optimal in these marshes sediments, which shows low bacterial diversity (Mesa et al., 2016). In these cases, increasing the bacterial biocatalyst activity offset the advantages of niche fitness (Vogel, 1996; Kuiper et al., 2004).

Plant growth partly depends on the accumulation of carbon not consumed during respiration. Abiotic stress may decrease growth and induce plants to raise respiration rates in order to fuel with ATP the maintenance processes. Thus, the present research is based on the hypothesis that beneficial root effect after bacterial bioaugmentation under heavy metal stress may be linked to a positive carbon balance at root level, related to absolute and/or relative alterations of COX and AOX pathways, as well as the decrease of ROS. It is known that increased respiration is a typical plant response to abiotic stress that may lead to increase ATP production (Jacoby et al., 2011, 2013). Also, the activity of AOX has been largely hypothesized to maintain ROS homeostasis under stress (Vanlerberghe, 2013). However, increased rates of total respiration are associated with higher consumption of carbon, leaving less carbon for growth, which does not favor to phytoremediation purposes. Recently, arbuscular mycorrhizal fungus inoculation has shown to decrease root respiration via COX pathway and increase plant growth (Romero-Munar et al., 2017). Continuing our focus on symbiotic relations, bacteria may also influence root respiration rate by the release of some compounds to the rhizosphere (Adesemoye and Egamberdieva, 2013). In this sense, some authors measured root respiration for bacterial inoculation, generally showing an increased root respiration rate (Hadas and Okon, 1987; Phillips et al., 1999; Zhou et al., 2015; Qin et al., 2016). However, the effect of rhizobacterial bioaugmentation in roots has not been tested considering the electron partitioning between the COX and AOX pathways, much less under heavy metal stress. According to results here presented, inoculated *S. maritima* showed a slower *in vivo* root respiration rate in heavy metal polluted sediments via both the COX and AOX pathways, with particular emphasis on the AOX pathway, which was not observed for mycorrhizal symbiosis (Romero-Munar et al., 2017). The decrease in total respiration suggests that rhizobacterial inoculation induces in roots much less carbon consumption for maintenance purposes. Contrary to what we observed in inoculated plants, the absence of inoculation is associated with faster respiration that ultimately may lead to increase both carbon consumption and respiratory ATP synthesis (Jacoby et al., 2011).

On the other hand, our results revealed that the slower respiration rate was accompanied with lower activities of antioxidant enzymes in bioaugmented *S. maritima* plants, suggesting improved plant tolerance under heavy metal stress after rhizobacterial inoculation (Ahemad, 2012; Mesa et al., 2015b). Some authors state that *Spartina* species possess a well organized and appropriately modulated antioxidative defense system that results in a normal plant development (Martínez Domínguez et al., 2009, 2010). Concretely, *S. maritima* has shown to have antioxidant feedback responses in the presence of heavy metals (Duarte et al., 2013; Van Oosten and Maggio, 2015), but its enzyme response in polluted scenarios after PGPR inoculation was unknown prior to this study. In this work, rhizobacterial bioaugmentation lessened CAT, SOD, and GPX activities in *S. maritima* roots compared to non-inoculated plants. This is interesting considering that root metal accumulation was higher after inoculation. Then, an increment in the generation of ROS, and consequently antioxidant enzyme activity, would be expected. Although several authors state that rhizobacteria mediates up-regulation of antioxidative enzymes (reviewed in Rajkumar et al., 2012), our data support that bacteria may contribute to the amelioration of abiotic stress not by modulating enzymatic activity, but reducing heavy metal toxicity. For example, siderophores released by the rhizobacterial consortium used (Mesa et al., 2015a) are chelators that may bind metals alleviating their toxicity (Dimkpa et al., 2009a,b). In the same way, selected rhizobacteria produce indoleacetic acid (IAA) (Mesa et al., 2015a), which has a bioprotective effect. Besides, respiration via AOX is thought to play an important role facing the stress induced by metal toxicity and prevents the generation of ROS (Lösch, 2004; Keunen et al., 2011), but our results showed that this pathway was greatly reduced in inoculated *S. maritima*. Collectively, these findings suggest that rhizobacterial inoculation induced a decrease in the formation of cell damaging free radicals, thus reducing the need of plant enzymatic defenses (Dimkpa et al., 2009a,b) and may also explain the lessened root activity of AOX in inoculated roots. In non-inoculated plants, greater activities of antioxidant enzymes and AOX may indicate a higher *S. maritima* sensitivity to heavy metal stress, and such phenomena could contribute to maintain ROS homeostasis.

After PGPR inoculation, the physiological adjustments mentioned above were accompanied by an increase in plant soluble protein content, which generally reflects a good plant physiological status (Gepstein, 1988). In shoots, this fact could be related with the amelioration of photosynthetic parameters observed in our previous research (Mesa et al., 2015b), given the likely importance of some proteins quantity, such as Rubisco, in determining plant photosynthetic capacity (Sybesma, 1983; Gepstein, 1988). Although photosynthesis and respiration are rarely studied together, it is advisable because their pathways are intertwined to constitute the entire bioenergetic plant machinery (Lambers and Ribas-Carbó, 2005). In our previous work, inoculation with the rhizobacterial consortium under the same experimental conditions had a beneficial effect on the photosynthetic apparatus of *S. maritima*, reflected in terms of functionality of PSII, values of *F_v_/F_m_* and Φ_PSII_ or chlorophyll pigments (Mesa et al., 2015b). Altogether, increased ATP and carbon availability may permit enhanced biomass formation in inoculated *S. maritima*, which would explain the increment in root growth in both this study and our previous one (Mesa et al., 2015b).

Results presented here are first findings at the organism level that open interesting hypotheses in plant biology. In summary, we suggest that inoculation of *S. maritima* plants with the native PGPR consortium decreased the activity of antioxidant enzymes and plant respiration, notably falling AOX pathway. Together with ameliorated photosynthesis results obtained in a previous work under the same experimental conditions, it may be elucidated that such processes allow *S. maritima* to accumulate more carbon for root biomass formation and increase their heavy metal rhizoaccumulation capacity in polluted soils. More experiments are needed with a view to more specific mechanistic approaches, as well as it would be very interesting to know to what extent the structure of the soil microbiota differs between the inoculated versus non-inoculated treatments. Their elucidation may be highly relevant in heavy metal hyperaccumulator plants like *S. maritima*, given their biotechnological potential in environmental decontamination.

## Author Contributions

EM-N, JM-M, and ND-S designed the research. JM-M and ND-S performed the experiments and interpreted the data. JM-M and ND-S wrote the manuscript. EM-N, MR-C, SR-G, EP, and IR-L revised the manuscript.

## Conflict of Interest Statement

The authors declare that the research was conducted in the absence of any commercial or financial relationships that could be construed as a potential conflict of interest.
